# EQ-5D outcomes in adults with autoimmune hepatitis: A GRADE-assessed systematic review and meta-analysis

**DOI:** 10.1038/s41598-026-53378-7

**Published:** 2026-05-18

**Authors:** Nemani Sai Manasa, Parameswaran Karuppanan, Hariharan Murugadoss, Mothishwaran Bhuvaneshwaran, S. Jennie

**Affiliations:** 1 Department of General Medicine, SRM Medical College Hospital and Research Centre, Faculty of Medicine and Health Sciences, SRM Institute of science and technology, Kattankulathur, Chengalpattu, Tamilnadu India; 2 Department of Pharmacy practice, Faculty of Medicine and Health Sciences, SRM College of Pharmacy, SRM Institute of Science and Technology, Kattankulathur, Chengalpattu, Tamilnadu India; 3 Directorate of Research, Faculty of Medicine and Health Sciences, Interdisciplinary Institute of Indian System of Medicine, SRM Institute of Science and Technology, Kattankulathur, Chengalpattu, Tamilnadu India

**Keywords:** Autoimmune Hepatitis, AIH, Health-related Quality of life, EQ-5D, HTA, Diseases, Gastroenterology, Health care, Immunology, Medical research

## Abstract

Autoimmune hepatitis (AIH) is a chronic immune-mediated liver disease that can substantially affect health-related quality of life (HRQoL). Although EQ-5D is widely used as a generic preference-based HRQoL instrument, EQ-5D outcomes in adults with AIH have not previously been synthesized in a focused systematic review. This review aimed to summarize EQ-5D outcomes in adults with AIH and assess the certainty of the available evidence using GRADE. A systematic review and meta-analysis was conducted in accordance with PRISMA and prospectively registered in PROSPERO (CRD420261334849). PubMed/MEDLINE, Scopus, Embase, and CENTRAL were searched from inception to 25 February 2026. Studies reporting EQ-5D utility/index, EQ-VAS, and/or domain-level outcomes in adults with AIH were included. Qualitative synthesis served as the primary interpretive framework, and quantitative synthesis was performed as an exploratory summary when appropriate. Certainty of evidence was assessed using GRADE. Five studies were included. Reported EQ-5D utility values ranged from 0.65 to 0.88, and EQ-VAS values ranged from 67.5 to 77.3 across studies. Exploratory random-effects synthesis yielded a pooled EQ-5D utility estimate of 0.80 (95% CI: 0.71 to 0.89; I² = 99.3%) and a pooled EQ-VAS estimate of 70.87 (95% CI: 67.28 to 74.46; I² = 93.9%). However, these pooled values should be interpreted as descriptive summaries of a highly heterogeneous evidence base rather than as stable benchmark estimates. Domain-level data from two studies suggested that pain/discomfort and anxiety/depression were the most frequently affected EQ-5D dimensions. Certainty of evidence was very low for all synthesized outcome groups. Available EQ-5D evidence in adults with AIH suggests an important HRQoL burden, particularly in the pain/discomfort and anxiety/depression domains. However, the evidence base is sparse, highly heterogeneous, and of very low certainty; therefore, pooled EQ-5D and EQ-VAS values should be interpreted as exploratory descriptive summaries rather than definitive benchmark estimates.

**Supplementary Information:**

The online version contains supplementary material available at 10.1038/s41598-026-53378-7.

## Introduction

Autoimmune hepatitis (AIH) is a chronic immune-mediated inflammatory liver disease that can lead to progressive liver injury, cirrhosis, liver failure, and the need for transplantation if not recognized and treated appropriately^1–3^. Although considered a relatively uncommon disease, its clinical burden is important because AIH often requires long-term follow-up, prolonged immunosuppressive therapy, and continuous monitoring to maintain remission and prevent disease progression^2,4^.

In recent years, there has been increasing recognition that clinical and biochemical outcomes alone are not sufficient to capture the full burden of AIH. Even when disease control is achieved, many patients continue to experience limitations in everyday well-being, including physical, emotional, and social impairment. For this reason, health-related quality of life (HRQoL) has become an important patient-centered outcome in the evaluation of chronic liver diseases, including AIH^5,6^.

The EuroQol 5-Dimension (EQ-5D) instrument is one of the most widely used generic tools for measuring HRQoL. It is particularly valuable because it provides both a descriptive health profile and a preference-based utility value that can be used for clinical interpretation, cross-disease comparison, and health-economic evaluation^7,8^. Although a range of HRQoL instruments has been used in AIH, including SF-36 and liver disease-specific questionnaires, EQ-5D was chosen as the focus of this review because it offers a standardized and comparable metric for quantitative synthesis across studies and populations^9^. Given the heterogeneity of HRQoL instruments used in the AIH literature, restricting the review to EQ-5D allowed a more uniform synthesis of the available evidence.

Despite the increasing use of HRQoL measures in AIH research, the available evidence on EQ-5D outcomes in adults with AIH remains limited and scattered. Individual studies have reported EQ-5D findings, but these data have not been comprehensively synthesized in a focused systematic review. As a result, the overall magnitude of HRQoL impairment in adults with AIH, the consistency of reported EQ-5D outcomes, and the certainty of the available evidence remain unclear. This represents an important gap, because a clearer summary of EQ-5D outcomes could improve understanding of patient burden and support future research, clinical decision-making, and health-economic assessment.

Therefore, a systematic review and meta-analysis dedicated specifically to EQ-5D outcomes in adults with AIH is needed. In addition, applying the GRADE (Grading of Recommendations Assessment, Development and Evaluation) approach is important to determine how much confidence can be placed in the available evidence, particularly in a field where studies may be few and heterogeneous^10^. Accordingly, the objective of this study is to systematically review and quantitatively synthesize EQ-5D outcomes in adults with autoimmune hepatitis and to assess the certainty of the evidence using GRADE.

## Methodology

### Study design and reporting framework

This systematic review and meta-analysis were conducted in accordance with the Preferred Reporting Items for Systematic Reviews and Meta-Analyses (PRISMA) guidelines^11,12^. The review protocol was prospectively registered in the International Prospective Register of Systematic Reviews (PROSPERO) under registration number (CRD420261334849)^13^. A completed PRISMA checklist was prepared to ensure transparent and comprehensive reporting of the review process **(Supplementary Fig. 12)**.

### Search strategy

A comprehensive literature search was undertaken in PubMed/MEDLINE, Scopus, Embase, and the Cochrane Central Register of Controlled Trials (CENTRAL) to identify potentially relevant studies. The search was performed from database inception to 25 February 2026. The strategy was developed using key concepts related to the population and outcomes of interest. Terms related to autoimmune hepatitis were combined with terms related to EQ-5D, including EQ-5D utility, EQ-5D index, EQ-5D visual analogue scale (VAS), and related health utility terminology **(Supplementary Tables 1–4)**. Controlled vocabulary and free-text keywords were used where appropriate, and the strategy was adapted to the indexing structure of each database. In addition, the reference lists of all included studies and relevant review articles were manually screened to identify any additional eligible publications that may have been missed through electronic database searching. Dedicated grey literature sources and trial registries were not systematically searched.

### Eligibility criteria

Studies were considered eligible if they included adult participants aged 18 years or older with a diagnosis of autoimmune hepatitis and reported EQ-5D outcomes, including EQ-5D utility or index scores, EQ-5D VAS scores, and/or responses at the individual domain level. Observational studies, including cross-sectional, case-control, and cohort designs, as well as randomized controlled trials, were considered eligible provided that the latter reported relevant baseline pre-intervention EQ-5D data. Only full-text articles published in English were included. Studies were excluded if they were case reports, case series, conference abstracts, editorials, letters, commentaries, narrative reviews, systematic reviews, or meta-analyses. Studies involving pediatric populations, studies not reporting extractable EQ-5D outcomes for AIH, and studies in which AIH data could not be separated from other liver diseases or autoimmune conditions were also excluded.

### Study selection

All retrieved records were exported to citation management software, and duplicates were removed prior to screening. The remaining records were then imported into the Rayyan web platform to facilitate study selection^14^. Title and abstract screening was performed independently by at least two reviewers. Full texts of potentially relevant studies were then assessed independently for eligibility. Any discrepancies arising during title/abstract screening or full-text review were resolved through discussion and, where necessary, consultation with a third reviewer (JS). The final set of included studies was determined by consensus.

### Data extraction

A structured data extraction form was developed in Microsoft Excel 365 prior to formal extraction. To improve consistency and methodological rigor, the extraction sheet was pilot-tested on a small number of studies and refined before full data collection began. Data extraction was performed independently by two reviewers (SM and PK) and cross-checked by a third reviewer (HM) for accuracy and completeness. From each included study, data were extracted on the author and year of publication, country and study setting, study design, and sample size. Participant-related variables were also collected, including age, sex distribution, diagnostic criteria used for autoimmune hepatitis, disease duration, remission status, cirrhosis status, and other relevant clinical characteristics where available. In addition, details relating to the EQ-5D instrument were recorded, including the version used (such as EQ-5D-3 L or EQ-5D-5 L), EQ-5D utility or index scores, EQ-5D VAS scores, and the proportions of participants reporting problems across individual EQ-5D domains whenever such data were available. Where reported, we also extracted the tariff or value set used to derive EQ-5D utility scores. Because value-set differences were expected to affect cross-study comparability, this information was extracted specifically to support interpretation of heterogeneity and indirectness in the quantitative synthesis. Outcome data were extracted as measures of central tendency and dispersion, including means and standard deviations, or medians with interquartile ranges and related summary statistics where applicable. When outcome data were incomplete or not presented in a directly usable format, attempts were made to derive or approximate the required summary estimates using accepted statistical methods, provided that sufficient data were available^15,16^.

### Risk of bias assessment

The methodological quality of the included studies was assessed independently by two reviewers (PK and HM), with disagreements resolved through discussion or, when required, consultation with a third reviewer (JS). Piloting of the quality assessment and refinement of the data extraction form were carried out simultaneously using the same five randomly selected studies.

For cross-sectional studies, the AXIS tool was used to evaluate methodological quality^17^. AXIS was selected because it is specifically designed for cross-sectional studies and captures key domains relevant to this evidence base, including clarity of study aims, appropriateness of sampling, validity of measurement, handling of non-response, and transparency of reporting. For the single prospective cohort study, the cohort version of the Newcastle–Ottawa Scale (NOS) was applied^18^. Because this study contributed descriptive HRQoL outcome data rather than comparative exposure-effect estimates, the NOS was applied in an adapted manner, with emphasis placed on participant selection, outcome ascertainment, adequacy of follow-up, and adjustment for relevant clinical covariates where reported. The comparability domain was interpreted according to whether important prognostic characteristics were accounted for analytically, rather than through strict comparison between exposed and non-exposed groups. These tools were selected to provide study-design-appropriate appraisal of internal validity, sampling methods, outcome measurement, and reporting quality across the included evidence base.

### Certainty of evidence

The certainty of evidence for each synthesized outcome was assessed using the GRADE framework in GRADEpro^19^. Because this review synthesized descriptive HRQoL outcomes rather than intervention effects, GRADE was applied to evaluate confidence in the estimated EQ-5D outcome values and summaries, rather than in comparative treatment effects. Certainty was assessed across the standard GRADE domains of risk of bias, inconsistency, indirectness, imprecision, and potential publication bias. Evidence was downgraded when important methodological limitations, substantial heterogeneity, limited comparability of populations or outcome measurement, wide confidence intervals, or possible reporting biases reduced confidence in the observed estimates. Certainty ratings were categorized as high, moderate, low, or very low and were used to indicate the level of confidence that could be placed in the available evidence for each synthesized outcome. The certainty of evidence, as assessed using the GRADE approach, is summarized in **Supplementary Fig. 11**.

### Outcome measures

The primary outcomes of interest were EQ-5D utility or index scores and EQ-5D VAS scores. Secondary outcomes included the proportion of participants reporting problems in the individual EQ-5D domains of mobility, self-care, usual activities, pain/discomfort, and anxiety/depression. Where domain-level data were available, responses reflecting any degree of impairment were grouped as “problem present,” whereas responses indicating no impairment were categorized as “no problem.”

### Data synthesis and statistical analysis

A qualitative synthesis was undertaken for all included studies and served as the primary interpretive framework for this review. Study characteristics, EQ-5D instrument versions, value sets/tariffs, and reported outcome estimates were summarized descriptively to facilitate direct comparison across studies. Quantitative synthesis was additionally performed when at least two studies reported sufficiently comparable EQ-5D summary data for the same outcome. All statistical analyses were performed in R (R Foundation for Statistical Computing, Vienna, Austria), and the meta-analyses were conducted using the metafor package.

A key methodological consideration was that EQ-5D-3 L and EQ-5D-5 L utility scores derived using different national value sets are not directly interchangeable, and this lack of harmonization was expected a priori to limit comparability across studies. Accordingly, pooled utility estimates were interpreted cautiously as exploratory summaries of published study-level values, rather than as fully harmonized or directly comparable estimates. Subgroup analysis by EQ-5D version was undertaken only as an exploratory assessment of one potential source of heterogeneity. Quantitative pooling was therefore retained as a secondary descriptive exercise to summarize the central tendency and dispersion of reported study-level EQ-5D estimates across the limited evidence base, rather than to derive a single clinically generalizable benchmark value for adults with AIH.

For continuous outcomes, including EQ-5D utility/index scores and EQ-VAS scores, exploratory pooled mean estimates were calculated using reported means and standard deviations. Where studies reported medians and dispersion measures instead of means and standard deviations, these were converted using established methods when appropriate. Separate random-effects meta-analyses were performed for EQ-5D utility/index scores and EQ-VAS scores. For EQ-5D domain-level outcomes, study findings were first synthesized narratively to describe the pattern of impairment across domains. Because only two studies contributed domain-level data, any corresponding quantitative summaries were treated only as exploratory two-study descriptive summaries intended to complement the narrative synthesis, rather than as definitive pooled prevalence estimates. Forest plots were generated to display individual study estimates and the extent of between-study dispersion.

Statistical heterogeneity was assessed using Cochran’s Q, I², and τ², and was interpreted alongside 95% prediction intervals. Consistent with Cochrane guidance, I² values were treated as descriptive indicators rather than rigid decision thresholds. A random-effects model was specified a priori, with between-study variance estimated using the restricted maximum likelihood (REML) method^20^, because true effects were not expected to be identical across studies. However, we recognized that important clinical and methodological heterogeneity could limit the interpretability of any pooled mean. Therefore, the meta-analyses were retained only as exploratory, model-based summaries of the distribution of observed study estimates, rather than as attempts to define a single clinically representative EQ-5D value for adults with autoimmune hepatitis. Accordingly, interpretation of the quantitative synthesis was based not only on the pooled estimates, but also on the range of observed study values, the magnitude of τ², the width of the prediction intervals, subgroup findings, leave-one-out sensitivity analyses, and the accompanying narrative synthesis. When heterogeneity was extreme or prediction intervals were very wide, greater interpretive weight was given to the individual study results and descriptive comparison across studies than to the pooled mean itself. In this review, pooled values were therefore considered secondary summaries of a highly dispersed evidence base and were not interpreted as stable benchmark estimates for clinical or economic decision-making.

Subgroup analysis according to EQ-5D instrument version was undertaken to explore whether use of EQ-5D-3 L versus EQ-5D-5 L contributed to between-study variability. Leave-one-out sensitivity analyses were also performed for the primary continuous outcomes to assess the influence of individual studies on the pooled estimates. Meta-regression for age and disease duration, as well as assessment of publication bias using Egger’s test, were prespecified but were not performed because the small number of included studies did not permit reliable analysis.

## Results

**Fig. 1:**
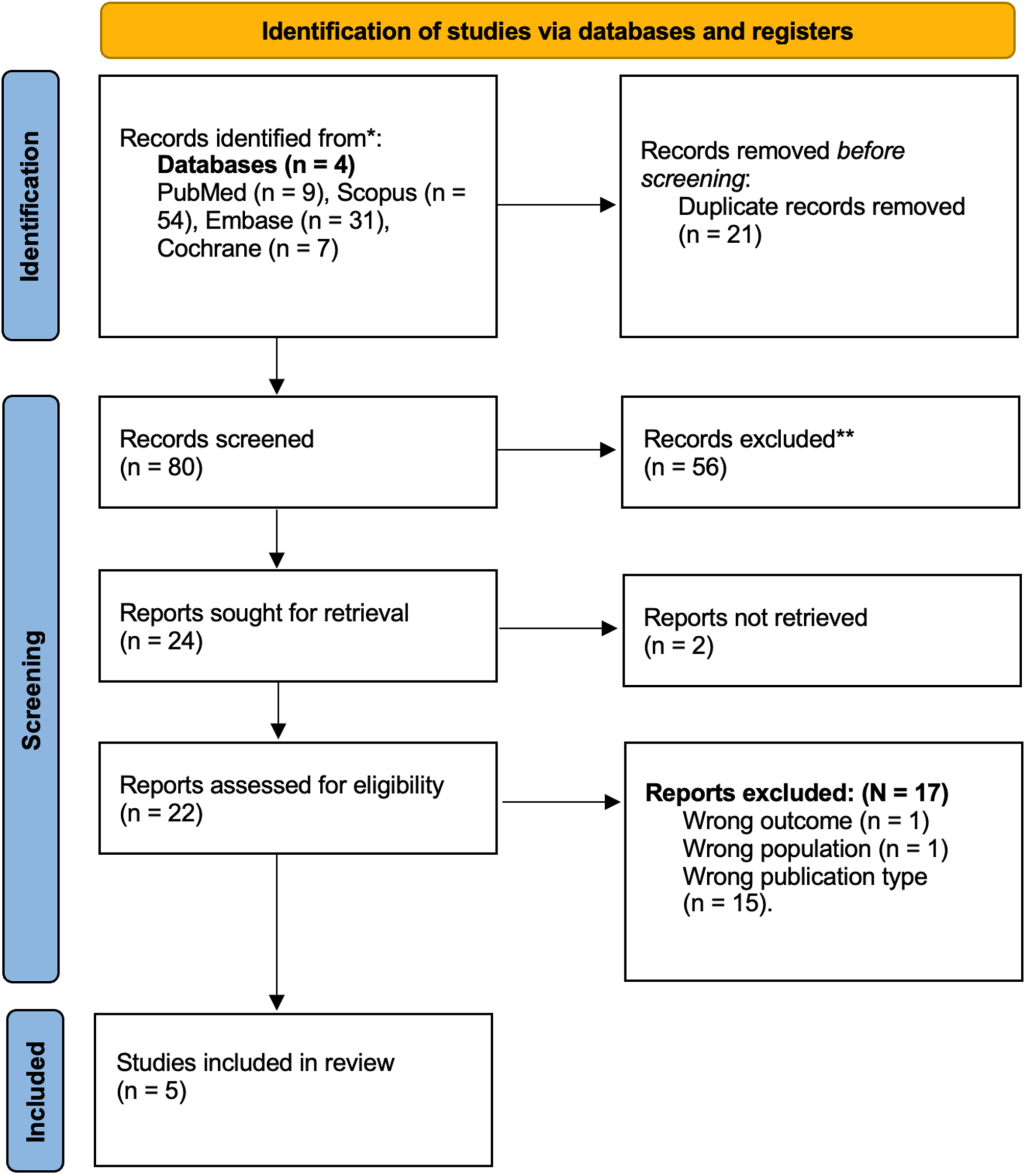
PRISMA Flowchart.

The electronic database search identified 101 records across four databases, including PubMed (*n* = 9), Scopus (*n* = 54), Embase (*n* = 31), and Cochrane (*n* = 7). After removal of 21 duplicate records, 80 studies underwent title and abstract screening. Of these, 56 records were excluded, and 24 reports were sought for retrieval. Two reports could not be retrieved, leaving 22 full-text articles assessed for eligibility. Following full-text review, 17 studies were excluded, including those with the wrong outcome (*n* = 1), wrong population (*n* = 1), and wrong publication type (*n* = 15). Ultimately, five studies were included in the systematic review, and all five contributed to the quantitative synthesis of EQ-5D utility and VAS outcomes. The study selection process is shown in Fig. 1, and the completed PRISMA 2020 checklist is provided in **Supplementary Fig. 12** to ensure transparent and comprehensive reporting of the review. The included studies represented a combined sample of 1,853 participants for the meta-analysis of EQ-5D utility/index scores and 1,852 participants for the meta-analysis of EQ-5D VAS scores. The studies were conducted across multiple settings, including the United Kingdom, Germany, Italy, and the United States. Most studies enrolled adults with autoimmune hepatitis exclusively, while one study was derived from a broader chronic liver disease cohort in which AIH-specific EQ-5D summary data were extractable and could therefore be included in the review. The included literature comprised cross-sectional, survey-based, and prospective observational studies, and three studies used the EQ-5D-5 L instrument, while two studies used the EQ-5D-3 L version **(**Table 1**)**.

Risk of bias was assessed for all five included studies using study-design-specific appraisal tools. Four studies with cross-sectional EQ-5D data were evaluated using the AXIS tool, while the single prospective cohort study was assessed using the cohort version of the NOS, applied in an adapted manner appropriate to descriptive observational data. Overall, the methodological quality of the evidence base was judged to be predominantly moderate, with no study considered unequivocally low risk of bias. Among the four studies assessed with AXIS, Michel et al. (2021), Wong et al. (2018), and Cortesi et al. (2020) were judged to have moderate risk of bias, whereas Wunsch et al. (2023) was considered to have high risk of bias. The most frequent concerns across the cross-sectional studies related to the lack of formal sample size justification, limited representativeness of the source population, and insufficient reporting of response rates or non-responder characteristics. These issues were particularly relevant in clinic-based studies and in studies where AIH-specific data were extracted from broader autoimmune or chronic liver disease cohorts. In contrast, most studies clearly stated their objectives, used appropriate outcome measures, employed validated EQ-5D instruments, and described their statistical methods adequately.

The prospective cohort study by Sierra et al. (2025) received 7 out of 9 stars on the adapted NOS, corresponding to a moderate risk of bias. Strengths of this study included prospective recruitment, clinically ascertained diagnoses, repeated outcome assessment, and multivariable adjustment for important covariates. However, the overall rating was limited by its single-center design, incomplete reporting of follow-up completeness, and the resulting constraints on external validity.

Taken together, these findings indicate that the current evidence on EQ-5D outcomes in adults with autoimmune hepatitis is based largely on studies with moderate methodological limitations, with the main threats to validity arising from sampling and selection issues rather than outcome measurement. Measurement bias was generally less concerning, as the included studies used established EQ-5D instruments and reported analyses with reasonable clarity. A summary of the risk of bias assessments is presented in **Supplementary Figs. 1 and 2**.

**Table 1:** Characteristics of Included studies.

Study label	Study design	Country/Setting	Sample size	Mean Age (SD)	Cirrhosis *n* (%)	EQ-5D version (3 L/5L)	Mean EQ-5D (SD)	Mean EQ-5D VAS (SD)	Tariff/value set used for EQ-5D
**Sierra L et al. 2025** (21)	Non-interventional, prospective, single-center cohort study	USA	230	52.1 (15.3)	60 (20.1)	3 L	0.88 (0.15)	77.28 (17.58)	NR
**Wong LL et al. 2018** (22)	Cross-sectional cohort study	UK	986	NR	NR	5 L	0.65 (0.18)	67.5 (14)	EQ-5D-5 L Value Set for England
**Michel M et al. 2021** (23)	Non-interventional cross-sectional cohort study (prospectively enrolled)	Germany	116	53.3 (17.04)	11 (9.5)	5 L	0.86 (0.18)	71.2 (20.5)	EQ-5D-5 L value set for Germany
**Cortesi PA et al. 2020** (24)	Observational, longitudinal, prospective, multicenter study	Italy	51	59 (13)	NR	3 L	0.871 (0.112)	68 (19.1)	Italian-specific social tariffs
**Wunsch E et al. 2023** (25)	Cross-sectional online survey	Multinational Europe (15 European countries)	470	NR	NR	5 L	0.75 (0.21)	69.95 (17.74)	NR

In Cortesi et al. (2020), the EQ-5D utility and EQ-VAS values included in this review corresponded to the AIH subgroup extracted from the broader chronic liver disease cohort. While subgroup-specific sample size and mean age were available, other clinical characteristics, including cirrhosis status, were not separately reported in extractable form for the AIH subgroup.

*NR – Not reported.

### Results of Meta-analysis

#### Overall EQ-5D score

**Fig. 2:**
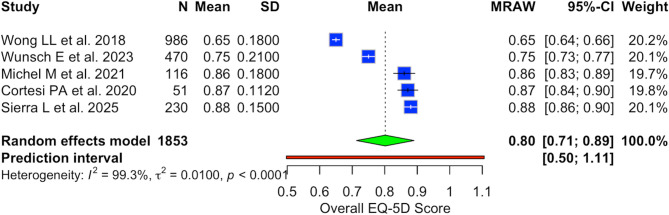
Forest Plot of Pooled EQ-5D Scores in Adults with Autoimmune Hepatitis.

Five studies comprising 1,853 adults with AIH reported overall EQ-5D utility values^21–25^, with individual study means ranging from 0.65 to 0.88. Because these estimates showed marked between-study dispersion, the qualitative interpretation of the study-level findings was prioritized. An exploratory random-effects synthesis yielded a summary mean of 0.80 (95% CI: 0.71 to 0.89), but heterogeneity was extreme (I² = 99.3%, τ² = 0.0100, *p* < 0.0001), and the prediction interval was very wide (0.50 to 1.11). Accordingly, this pooled value should not be interpreted as a single representative EQ-5D utility estimate for adults with AIH, but only as a model-based summary of highly heterogeneous study results **(**Fig. 2**)**.

In subgroup analysis according to EQ-5D instrument version, studies using the EQ-5D-5 L reported a pooled score of 0.83 (95% CI: 0.75 to 0.91), whereas studies using the EQ-5D-3 L showed a pooled score of 0.76 (95% CI: 0.54 to 0.98). Heterogeneity remained high within both subgroups (5 L: I² = 97.9%; 3 L: I² = 99.4%) **(Supplementary Fig. 3)**. The test for subgroup differences was not statistically significant (*p* = 0.5546); however, this analysis was based on only two studies in the EQ-5D-3 L subgroup and three studies in the EQ-5D-5 L subgroup and was therefore underpowered for reliable inference. Accordingly, the absence of a statistically significant subgroup difference should not be interpreted as evidence that instrument version or valuation approach did not contribute to heterogeneity.

Leave-one-out sensitivity analysis demonstrated that the pooled EQ-5D estimate remained broadly stable, ranging from 0.78 (95% CI: 0.68 to 0.88) after omitting Sierra et al. (2025) to 0.84 (95% CI: 0.78 to 0.90) after omitting Wong et al. (2018). Although omission of individual studies produced only modest changes in the pooled estimate, heterogeneity remained persistently high across all iterations (I² range: 97.1% to 99.5%), indicating that no single study alone accounted for the substantial between-study variability **(Supplementary Fig. 4)**.

Taken together, these findings indicate that the literature currently supports only a descriptive range of reported EQ-5D utility values rather than a stable pooled benchmark.

### Overall EQ-5D VAS score

**Fig. 3:**
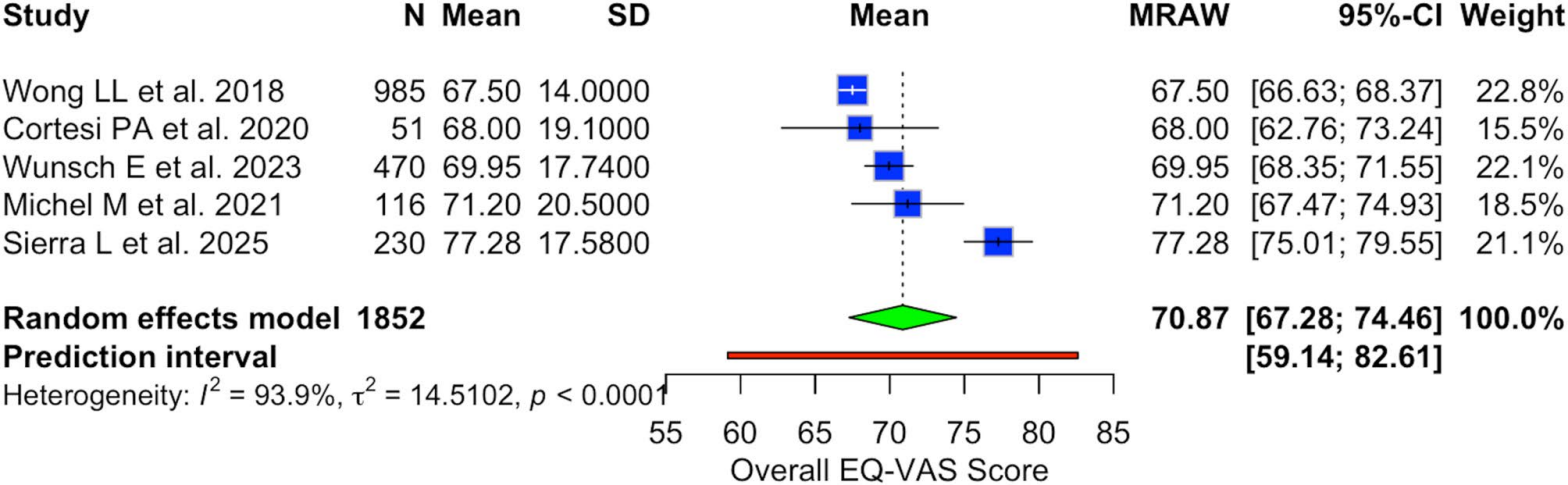
Forest Plot of Pooled EQ-VAS Scores in Adults with Autoimmune Hepatitis.

Five studies comprising 1,852 adults with AIH reported EQ-VAS values^21–25^, with individual study means ranging from 67.5 to 77.3. Because these estimates showed substantial between-study dispersion, the qualitative interpretation of the study-level findings was prioritized. An exploratory random-effects synthesis yielded a summary EQ-VAS mean of 70.87 (95% CI: 67.28 to 74.46), but heterogeneity was considerable (I² = 93.9%). Accordingly, this pooled value should not be interpreted as a stable estimate of typical HRQoL in adults with AIH, but rather as a descriptive summary of a highly heterogeneous evidence base **(**Fig. 3**)**.

Leave-one-out sensitivity analysis showed that the pooled EQ-VAS estimate remained relatively stable after sequential omission of each study, ranging from 68.95 (95% CI: 67.12 to 70.79) after excluding Sierra et al. (2025) to 71.87 (95% CI: 67.86 to 75.89) after excluding Wong et al. (2018). Although exclusion of Sierra et al. (2025) reduced heterogeneity to some extent (I² = 68.5%), heterogeneity remained moderate to high across the other iterations (I² range: 89.8% to 95.4%), indicating that no single study fully accounted for the observed between-study variability **(Supplementary Fig. 5)**.

### Descriptive summary of EQ-5D domain-specific problems

Domain-level EQ-5D data were available from only two studies, Wong et al. (2018) and Cortesi et al. (2020)^22,24^, and were therefore interpreted primarily as descriptive findings rather than as definitive pooled prevalence estimates. Across both studies, the overall pattern was consistent: pain/discomfort and anxiety/depression appeared to be the most frequently affected domains, whereas self-care appeared to be the least affected. By contrast, limitations in mobility and usual activities were present in a smaller, although still clinically relevant, proportion of participants (Wong et al., 2018; Cortesi et al., 2020).

In the large UK cohort reported by Wong et al. (2018), 39% of participants reported at least some mobility problem, 25% reported problems with self-care, 42% reported limitations in usual activities, 58% reported pain/discomfort, and 43% reported anxiety/depression. A similar pattern was observed in the Italian cohort reported by Cortesi et al. (2020), in which the proportions reporting any problem were 25.5% for mobility, 13.7% for self-care, 23.5% for usual activities, 58.8% for pain/discomfort, and 51.0% for anxiety/depression. Taken together, these findings suggest that although basic self-care and mobility were relatively preserved for many patients, symptom burden and psychological distress were more prominent features of impaired HRQoL in the available AIH cohorts (Wong et al., 2018; Cortesi et al., 2020).

Consistent with this descriptive pattern, the corresponding exploratory two-study summary proportions were 0.58 (95% CI: 0.55 to 0.61) for pain/discomfort, 0.43 (95% CI: 0.40 to 0.46) for anxiety/depression, 0.38 (95% CI: 0.35 to 0.41) for mobility, 0.35 (95% CI: 0.23 to 0.49) for usual activities, and 0.24 (95% CI: 0.22 to 0.27) for self-care. However, because these estimates were derived from only two studies, they should be interpreted with substantial caution and regarded as provisional descriptive summaries rather than as stable or generalizable prevalence estimates for the wider AIH population **(Supplementary Fig. 6–10)**.

### GRADE assessment of certainty of evidence

GRADE assessment showed that the certainty of evidence was very low for all three synthesized outcome groups: overall EQ-5D utility, overall EQ-VAS, and EQ-5D domain-level summaries. This rating was mainly driven by the observational nature of the included studies, methodological limitations, substantial between-study heterogeneity, and the small number of studies with limited outcome data **(Supplementary Fig. 11)**.

## Discussion

This systematic review identified only five studies reporting EQ-5D outcomes in adults with AIH, and these studies showed substantial between-study variability in both utility and EQ-VAS values. The observed EQ-5D utility means ranged from 0.65 to 0.88, while EQ-VAS means ranged from 67.5 to 77.3. Because between-study heterogeneity was extreme, these study-level ranges were considered more informative than the pooled means. Accordingly, the principal contribution of this review lies not in deriving a single benchmark value, but in highlighting marked variability, sparse data, and important evidence gaps in the EQ-5D literature for AIH^22,23,26^.

When considered alongside the broader literature, our findings appear consistent with an established pattern of impaired HRQoL in autoimmune hepatitis. Previous studies have reported lower HRQoL among patients with AIH compared with healthy controls or general-population reference groups, with fatigue, psychological symptoms, and treatment burden identified as important contributors^5,26,27^. Similarly, the prior systematic review by Honoré et al. (2018)^28^ concluded that HRQoL is adversely affected in AIH, while Snijders et al. (2021)^29^ highlighted the importance of psychosocial burden, particularly anxiety and depression. More broadly, literature across chronic liver diseases indicates that symptom burden, uncertainty, and unmet supportive needs can substantially impair quality of life across disease etiologies^30^. Published general-population EQ-5D norms may offer only broad contextual reference points. For example, reported mean EQ-5D-5 L index values are 0.88 in Germany and 0.93 in Italy, while age-specific norms in the United States for adults aged 45 years and older range from 0.811 to 0.824. However, these comparisons are indirect and should not be interpreted as formal evidence of a decrement attributable to AIH, because the included studies did not include matched comparator groups and also differed from the reference datasets in country, age structure, EQ-5D version, and tariff/value set. Accordingly, these external comparisons are presented for context only rather than for inferential interpretation^30–32^. More fundamentally, the absence of internal comparator groups across the included studies represents a structural limitation of the evidence base and restricts interpretation to descriptive reporting of EQ-5D outcomes observed in AIH cohorts.

A major methodological issue in this review was the extreme heterogeneity observed in the quantitative syntheses of both EQ-5D utility and EQ-VAS. Under these conditions, the pooled estimates are not best understood as definitive single values, but rather as exploratory summaries of a broad distribution of study effects. Although the included studies assessed the same broad generic HRQoL construct in adults with autoimmune hepatitis, the random-effects model was used primarily to characterize the distribution and dispersion of study estimates across settings. Accordingly, interpretation relied not on the pooled mean alone, but also on the study-level range, τ², prediction intervals, subgroup analyses, leave-one-out sensitivity analyses, and the accompanying narrative synthesis. The prediction intervals were especially informative, showing that the expected range of effects across settings was wide. In particular, the EQ-5D utility prediction interval extended beyond the theoretical upper bound of the EQ-5D scale, which likely reflects model instability under extreme heterogeneity rather than plausible true values above 1. Under these conditions, the pooled EQ-5D utility estimate should not be interpreted as a clinically transferable benchmark or used directly for health-economic application. In this context, the value of the quantitative synthesis lies mainly in making the extent of inconsistency explicit through τ² and prediction intervals, whereas substantive interpretation depends primarily on the individual study estimates and their clinical context.

Among the included studies, Wong et al. (2018) contributed the largest sample (*n* = 986) and reported the lowest mean EQ-5D utility and EQ-VAS values in the review, while key study-level covariates relevant to heterogeneity assessment, including mean age and cirrhosis prevalence, were not reported in extractable summary form^22^. This limited assessment of comparability between that cohort and the remaining studies. In leave-one-out analyses, exclusion of Wong et al. modestly increased the pooled EQ-5D estimate to 0.84 and the pooled EQ-VAS estimate to 71.87, but substantial heterogeneity persisted, indicating that this study was influential but did not alone account for the observed between-study variability. Accordingly, the overall summary should still be interpreted cautiously in light of the incomplete reporting of covariates in the largest included study.

An additional interpretive consideration is the minimally important difference (MID/MCID) for EQ-5D outcomes. In some settings, published literature suggests that a difference of approximately 0.074 to 0.08 for the EQ-5D-3 L utility index and around 7 points for the EQ-VAS may be clinically meaningful. However, application of these thresholds to the present review is limited because the included studies reported mainly cross-sectional descriptive values rather than within-person changes, and because different EQ-5D versions, tariffs, and study populations were represented. MID/MCID values therefore provide only broad context and should not be used to over-interpret the pooled summaries reported here.

Several plausible explanations may account for the observed heterogeneity. First, the included studies differed in important clinical factors likely to influence HRQoL, including remission status, cirrhosis burden, disease duration, treatment exposure, and comorbidities. Previous AIH studies have suggested that these variables may be associated with worse patient-reported outcomes^22,27^. Second, methodological diversity was considerable, as the review included cross-sectional, survey-based, and prospective observational studies that differed in recruitment setting, sampling strategy, and sample size. Third, EQ-5D utility measurement was not fully harmonized across studies. Both EQ-5D-3 L and EQ-5D-5 L were used, and reported utility values were derived from different national tariffs/value sets where specified, while some studies did not report the value set used. Because the published articles provided only aggregate utility values rather than individual health-state profiles, recalculation to a common tariff or standardized crosswalk harmonization was not feasible. In addition, because the review relied solely on published reports and no additional unpublished value-set information was sought from study authors, missing tariff/value-set information in some studies could not be resolved further. This remains an additional source of indirectness and limited comparability across the pooled utility estimates. Together, these issues likely contributed to heterogeneity and further limit the interpretability of the pooled utility findings. Although subgroup analysis by EQ-5D version was performed, it included only two to three studies per subgroup and should therefore be regarded as exploratory rather than explanatory. Finally, one study contributed AIH-specific EQ-5D estimates extracted from a broader chronic liver disease cohort rather than from an AIH-only sample. Although the subgroup outcome values were extractable and therefore eligible for inclusion, subgroup-level clinical characterization was incomplete, which may have introduced additional interpretive variability.

The domain-level findings also require careful interpretation. Pain/discomfort appeared to be the most commonly affected EQ-5D dimension, followed by anxiety/depression, whereas self-care was the least affected. This pattern is clinically plausible and consistent with prior AIH literature suggesting that the burden of disease relates strongly to pain, fatigue, emotional distress, and chronic disease uncertainty rather than marked impairment in basic self-care^5,26,29^. However, only two studies contributed domain-level data. As such, these domain-level summaries are best regarded as exploratory descriptive findings rather than robust prevalence estimates for the wider AIH population.

Another important limitation is that the small number of studies and inconsistent reporting of study-level covariates prevented formal exploration of heterogeneity through meta-regression. In principle, factors such as age, disease duration, cirrhosis, remission status, corticosteroid exposure, immunosuppressive regimen, comorbidity burden, and EQ-5D version would all be relevant candidates for quantitative exploration, particularly because previous AIH studies suggest that several of these factors may influence HRQoL. However, with only five studies included in the primary meta-analyses, meta-regression would have been statistically underpowered and potentially misleading. This restricted our ability to determine which patient- or study-level characteristics may be driving lower EQ-5D outcomes in AIH. In addition, none of the included studies incorporated matched healthy-control or disease-control groups, which limits the ability to determine the magnitude of any HRQoL decrement relative to external populations.

The findings of this review should therefore be interpreted with caution. Most included studies were observational, and several had moderate methodological limitations, with the principal risk-of-bias concerns relating to sampling, representativeness, and incomplete reporting of non-response. In addition, the small evidence base, marked inconsistency, and sparse domain-level data reduce confidence in the precision, stability, and generalizability of the pooled estimates. This concern is reinforced by our GRADE assessment, which rated the certainty of evidence as very low for all three synthesized outcome groups - overall EQ-5D utility, overall EQ-VAS, and EQ-5D domain-level summaries, mainly because of risk of bias, substantial heterogeneity, indirectness, and limited outcome data. Accordingly, confidence in these summary estimates remains limited.

At the review level, some limitations should also be acknowledged. Only full-text articles published in English were included, which may have introduced language bias. In addition, dedicated grey literature sources such as trial registries, conference proceedings, and other non-indexed sources were not systematically searched, and conference abstracts were excluded by design because they generally provide limited methodological detail and insufficient extractable outcome data for reliable synthesis. This approach may have excluded potentially informative EQ-5D data and may have contributed to the small evidence base available for review. The number of eligible studies was also small, precluding reliable formal assessment of publication bias or small-study effects; accordingly, the possibility of selective publication cannot be excluded. In addition, the evidence was derived largely from clinic-based or observational cohorts, which may reduce external validity. Nevertheless, this review has several strengths. It was prospectively registered, conducted according to PRISMA principles, used a comprehensive search strategy, applied study-design-specific risk-of-bias tools, and focused specifically on EQ-5D, a preference-based instrument with clear relevance to both clinical interpretation and health-economic evaluation. Importantly, a further strength of this review is that it clearly delineates the current evidence gaps and the sparseness of available EQ-5D data in adults with AIH, thereby providing a rationale for future larger, methodologically robust studies capable of generating higher-quality and more clinically informative HRQoL evidence.

In conclusion, the available EQ-5D evidence in adults with autoimmune hepatitis indicates an important HRQoL burden within reported AIH cohorts, particularly in the pain/discomfort and anxiety/depression domains. However, the current evidence base is limited by a small number of studies, moderate methodological weaknesses, very low certainty of evidence, and extreme between-study heterogeneity. The pooled EQ-5D and EQ-VAS estimates should therefore be interpreted as exploratory descriptive summaries rather than definitive benchmark values, and they should not be used directly for clinical benchmarking or economic application until larger, better reported, and methodologically harmonized studies become available.

### AI use and acknowledgment statement

The authors acknowledge the use of Paperpal and QuillBot AI tools solely for language editing and improving manuscript clarity. No generative artificial intelligence tools were used in the conceptualization, analysis, or drafting of scientific content.

The authors gratefully acknowledge the financial support by SRM Medical College Hospital and Research Centre, Faculty of Medicine and Health Sciences, SRMIST, Kattankulathur for bearing the defrayed cost of publishing this article.

## Electronic Supplementary Material

Below is the link to the electronic supplementary material.

Supplementary Material 1

## Data Availability

All data generated or analysed during this study are included in this published article and its supplementary materials.
