# Supplementary material for: EQ-5D outcomes in adults with autoimmune hepatitis: A GRADE-assessed systematic review and meta-analysis
Source: Sci Rep. 2026 May 18;16:22447. doi: 10.1038/s41598-026-53378-7 (PMC13376598; doi:10.1038/s41598-026-53378-7)
Supplement: Supplementary file 1 — Supplementary Material 1 [file 41598_2026_53378_MOESM1_ESM.docx]

**Supplementary Table 1: Search strategy-Pubmed**

| **Framework** | **Search Terms** | **Search Hits as on**  **25/02/2026** |
| --- | --- | --- |
| P-Population | "autoimmune hepatitis" OR "auto-immune hepatitis" OR AIH OR "Hepatitis, Autoimmune"[Mesh] | 10,001 |
| I-Intervention | NA |  |
| C-Comparator | NA |  |
| O-Outcome | EQ-5D OR EQ5D OR (EQ 5-Dimension) OR (EQ 5D) OR Euroqol | 22,413 |
| P AND O | (("autoimmune hepatitis" OR "auto-immune hepatitis" OR AIH OR "Hepatitis, Autoimmune"[Mesh])) AND (EQ-5D OR EQ5D OR (EQ 5-Dimension) OR (EQ 5D) OR Euroqol) | 9 |

**Supplementary Table 2: Search strategy-Scopus**

| **Framework** | **Search Terms** | **Search Hits as on  25/02/2026** |
| --- | --- | --- |
| P-Population - #1 | "autoimmune hepatitis" OR "auto-immune hepatitis" OR AIH | 41,524 |
| I-Intervention | NA |  |
| C-Comparator | NA |  |
| O-Outcome - #2 | EQ-5D OR EQ5D OR (EQ 5-Dimension) OR (EQ 5D) OR Euroqol | 70,007 |
| P AND O | #1 AND #2 | 54 |

**Supplementary Table 3: Search strategy-Embase**

| **Framework** | **Search Terms** | **Search Hits as on  25/02/2026** |
| --- | --- | --- |
| P-Population #1 | ‘autoimmune hepatitis’ OR ‘auto-immune hepatitis’ OR ‘AIH’ | 24,379 |
| I-Intervention | NA |  |
| C-Comparator | NA |  |
| O-Outcome #2 | EQ-5D OR EQ5D OR (EQ 5-Dimension) OR (EQ 5D) OR Euroqol | 41,766 |
| P AND O | #1 AND #2 | 31 |

**Supplementary Table 4: Search strategy-Cochrane library**

| **Framework** | **Search Terms** | **Search Hits as on  25/02/2026** |
| --- | --- | --- |
| P-Population #1 | "autoimmune hepatitis" OR "auto-immune hepatitis" OR AIH | 448 |
| I-Intervention | NA |  |
| C-Comparator | NA |  |
| O-Outcome #2 | EQ-5D OR EQ5D OR "EQ 5D" OR "EQ 5-Dimension" OR EuroQol | 17,135 |
| P AND O | #1 AND #2 | 7 |

**Supplementary Table 5: Excluded studies**

| **S.No** | **Study Label** | **Study title** | **Reason for exclusion** |
| --- | --- | --- | --- |
| 1 | Snijders, R.J et al. 2021 | Health-related quality of life in autoimmune hepatitis | Wrong publication type |
| 2 | Zhou, T et al. 2021 | Health-Related Quality of Life in Patients With Different Diseases Measured With the EQ-5D-5L: A Systematic Review | Wrong publication type |
| 3 | Younossi, Z.M et al. 2026 | Measuring health-related quality of life and patient-reported outcomes in chronic liver disease | Wrong outcome |
| 4 | Tang, S et al. 2021 | Do Drug Accessibility and OOP Burden Affect Health-Related Quality of Life of Patients With Chronic Diseases? — EQ-5D-5L Evaluation Evidence From Five Districts in China | Wrong population |
| 5 | Merelli, E et al. 2026 | Unsupervised Machine Learning Reveals Distinct Quality-of-Life Clusters Across Autoimmune Liver Diseases | Wrong publication type |
| 6 | Amaris, N.R et al. 2025 | UNCERTAINTY AND HEALTH-RELATED QUALITY OF LIFE IN AUTOIMMUNE LIVER DISEASES: A DESCRIPTIVE ANALYSIS OF PATIENT-REPORTED OUTCOMES | Wrong publication type |
| 7 | Sierra, L et al. 2024 | PREDICTORS OF SYMPTOM SEVERITY AMONG AUTOIMMUNE LIVER DISEASE PATIENTS IN A SINGLE UNITED STATES CENTER | Wrong publication type |
| 8 | Snijders, R et al. 2023 | The impact of a complete biochemical response on health-related quality of life in patients with autoimmune hepatitis: a multicentre prospective cross-sectional study | Wrong publication type |
| 9 | Dixon, S et al. 2022 | QUALITY OF LIFE AND ITS ASSOCIATED FACTORS IN AUTOIMMUNE HEPATITIS: A CROSS-SECTIONAL STUDY | Wrong publication type |
| 10 | Ferrigno, B et al. 2021 | Patients with autoimmune hepatitis and poor medication adherence have a lower health-related quality of life. | Wrong publication type |
| 11 | Dixon, S et al. 2021 | Quality of life in patients with autoimmune hepatitis: A review of patient-reported outcome measures | Wrong publication type |
| 12 | Wong, L.L et al. 2017 | Exploring the impact of autoimmune hepatitis on health-related quality of life | Wrong publication type |
| 13 | Conti, S et al. 2015 | Models of the impact of major liver diseases on EQ-5D visual analogue scale and utility-index: Convergences and divergences | Wrong publication type |
| 14 | Cortesi, P.A et al. 2014 | A comparison between the health-related quality of life reported by the general population and by patients with major liver diseases | Wrong publication type |
| 15 | Cortesi, P.A et al. 2014 | The impact of liver disease on the health-related quality of life | Wrong publication type |
| 16 | Cortesi, P.A et al. 2013 | The impact of type of liver conditions on the patients' health related quality of life | Wrong publication type |
| 17 | Cortesi, P.A et al. 2013 | Health related quality of life in the major liver conditions | Wrong publication type |

| **Author** | **Q1** | **Q2** | **Q3** | **Q4** | **Q5** | **Q6** | **Q7** | **Q8** | **Q9** | **Q10** | **Q11** | **Q12** | **Q13** | **Q14** | **Q15** | **Q16** | **Q17** | **Q18** | **Q19** | **Q20** |
| --- | --- | --- | --- | --- | --- | --- | --- | --- | --- | --- | --- | --- | --- | --- | --- | --- | --- | --- | --- | --- |
| **Michel et al., 2021** | 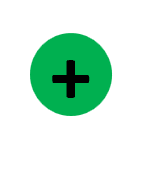 | 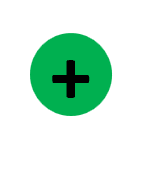 | 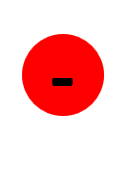 | 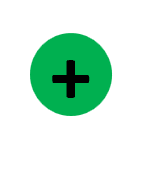 | 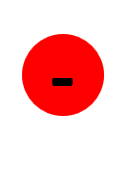 | 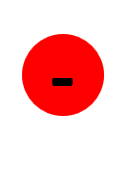 | 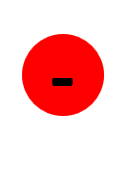 | 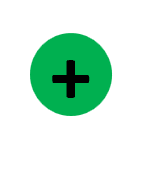 | 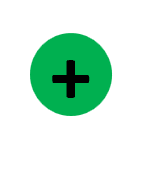 | 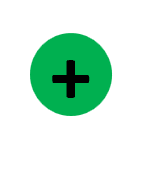 | 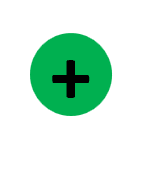 | 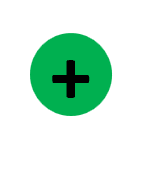 | 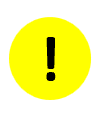 | 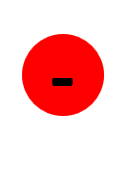 | 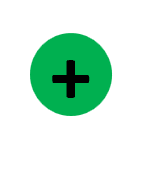 | 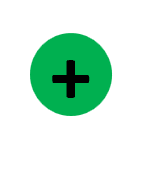 | 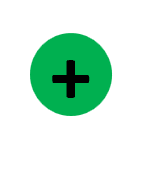 | 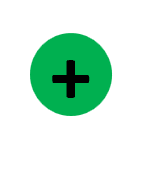 | 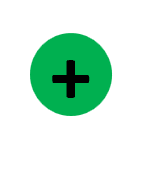 | 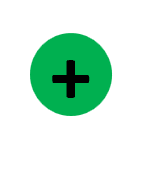 |
| **Wong et al., 2018** | 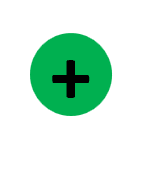 | 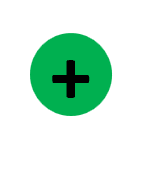 | 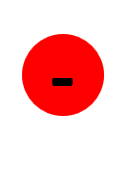 | 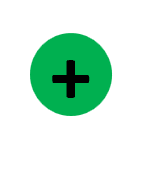 | 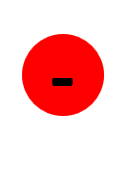 | 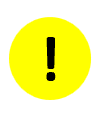 | 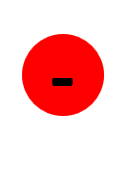 | 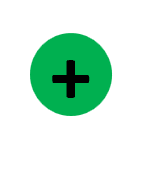 | 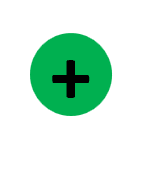 | 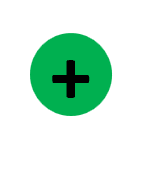 | 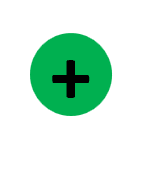 | 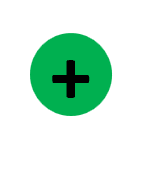 | 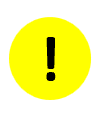 | 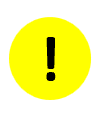 | 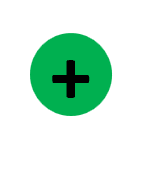 | 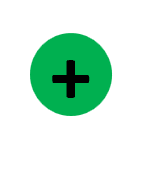 | 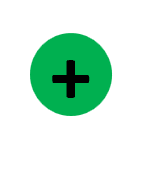 | 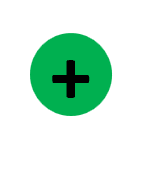 | 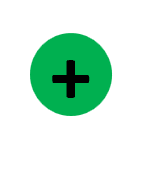 | 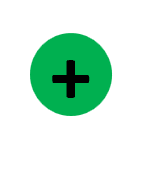 |
| **Cortesi et al., 2020** | 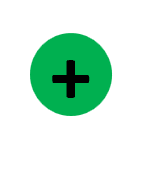 | 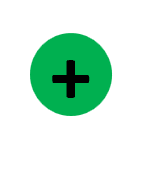 | 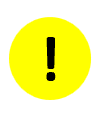 | 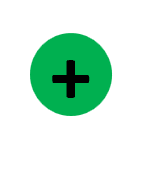 | 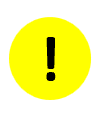 | 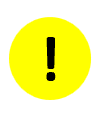 | 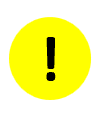 | 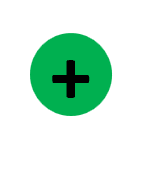 | 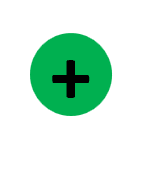 | 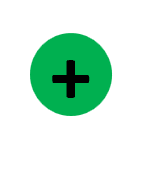 | 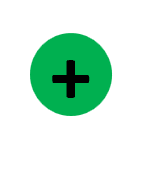 | 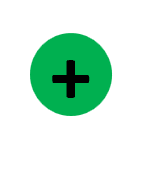 | 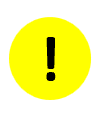 | 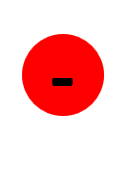 | 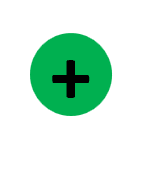 | 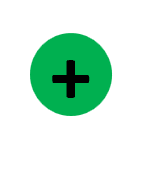 | 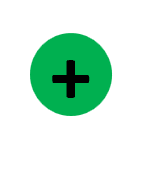 | 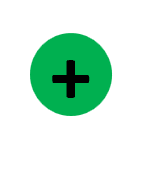 | 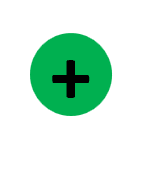 | 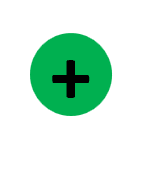 |
| **Wunsch et al., 2023** | 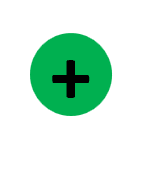 | 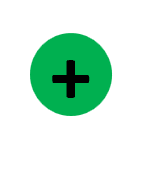 | 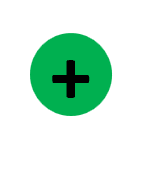 | 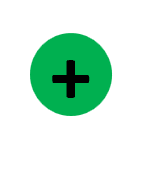 | 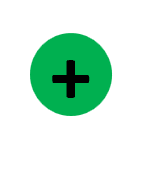 | 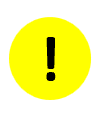 | 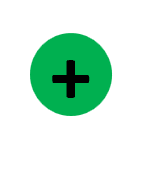 | 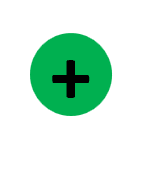 | 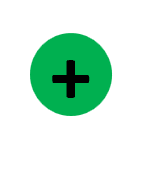 | 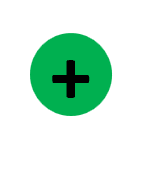 | 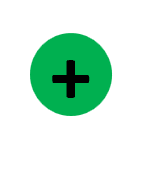 | 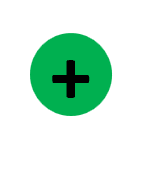 | 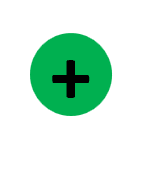 | 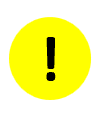 | 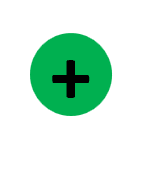 | 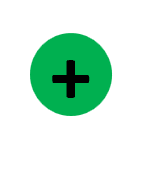 | 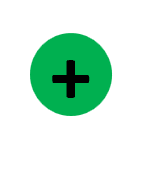 | 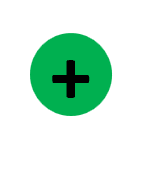 | 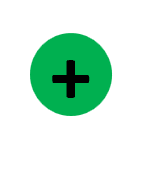 | 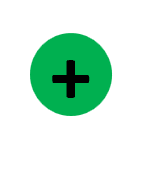 |

**Supplementary Figure 1. Risk of Bias assessment of included Cross-sectional studies using the AXIS tool.**

Q1. Were the aims/objectives of the study clear?

Q2. Was the study design appropriate for the stated aim(s)?

Q3. Was the sample size justified?

Q4. Was the target/reference population clearly defined? (Is it clear who the research was about?)

Q5. Was the sample frame taken from an appropriate population base so that it closely represented the target/reference population under investigation?

Q6. Was the selection process likely to select subjects/participants that were representative of the target/reference population under investigation?

Q7. Were measures undertaken to address and categorize non-responders?

Q8. Were the risk factor and outcome variables measured appropriate to the aims of the study?

Q9. Were the risk factor and outcome variables measured correctly using instruments/measurements that had been trialed, piloted or published previously?

Q10. Is it clear what was used to determine statistical significance and/or precision estimates (e.g., p-values, confidence intervals)?

Q11. Were the methods (including statistical methods) sufficiently described to enable them to be repeated?

Q12. Were the basic data adequately described?

Q13. Does the response rate raise concerns about non-response bias?

Q14. If appropriate, was information about non-responders described?

Q15. Were the results internally consistent?

Q16. Were the results presented for all the analyses described in the methods?

Q17. Were the authors’ discussions and conclusions justified by the results?

Q18. Were the limitations of the study discussed?

Q19. Were there any funding sources or conflicts of interest that may affect the authors’ interpretation of the results?

Q20. Was ethical approval or consent of participants attained?

| **Study label** | **Domain** | **NOS item** | **Stars** |
| --- | --- | --- | --- |
| **Sierra et al. (2025)** | Selection | Representativeness of the exposed cohort | **★** |
|  | Selection | Selection of the non-exposed cohort | **★** |
|  | Selection | Ascertainment of exposure | **★** |
|  | Selection | Outcome of interest not present at start |  |
|  | Comparability | Comparability on the basis of design/analysis | **★★** |
|  | Outcome | Assessment of outcome | **★** |
|  | Outcome | Was follow-up long enough for outcomes to occur? | **★** |
|  | Outcome | Adequacy of follow-up |  |
|  | **Total adapted NOS stars: 7/9 (moderate risk of bias)** | | |

**Supplementary Figure 2: Risk of Bias assessment of included Cohort study using the Newcastle-Ottawa scale.**

| 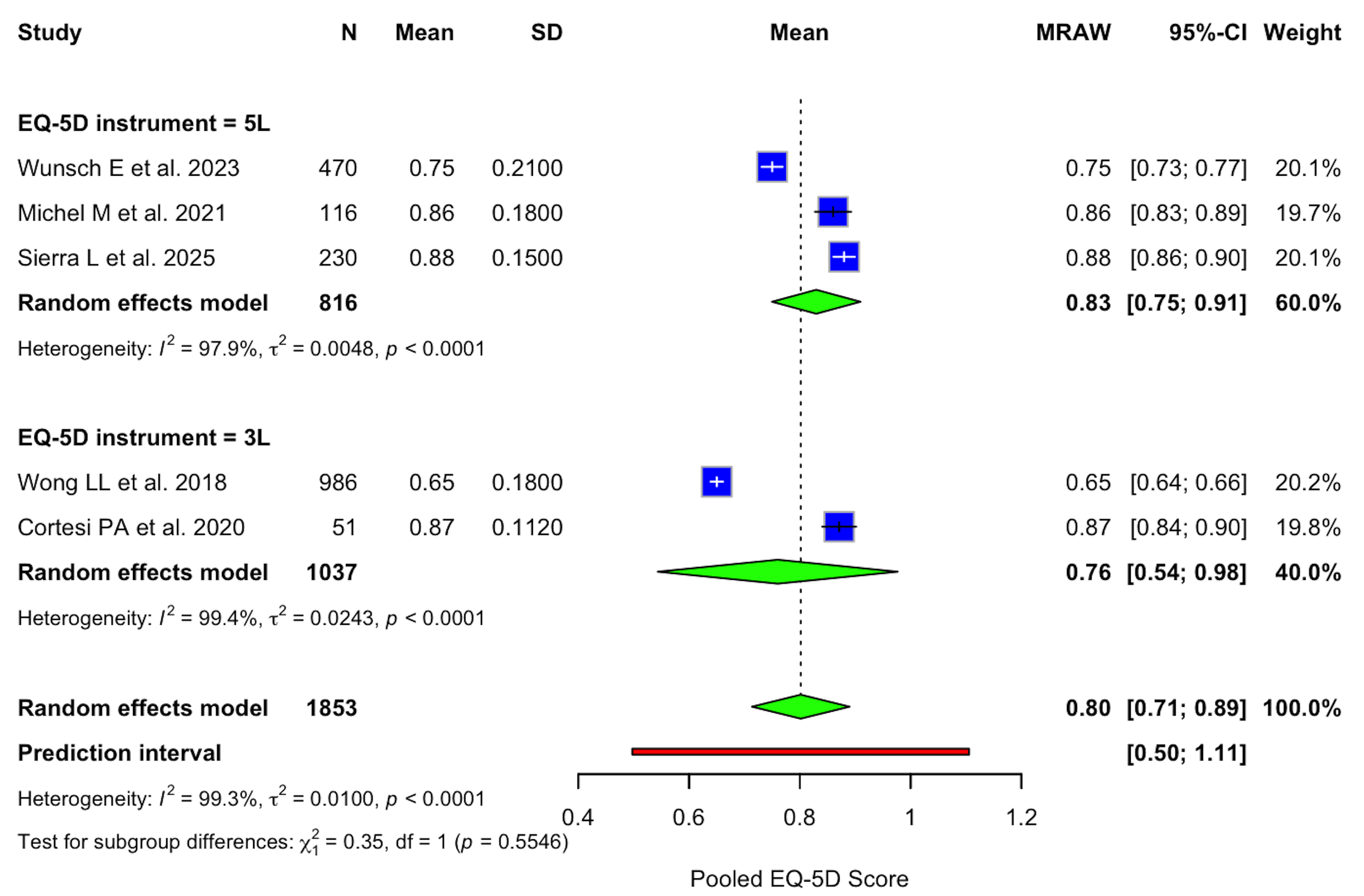 |
| --- |
| **Supplementary Figure 3. Subgroup analysis of pooled EQ-5D utility scores in adults with autoimmune hepatitis according to EQ-5D instrument version (3L vs 5L).** |

| 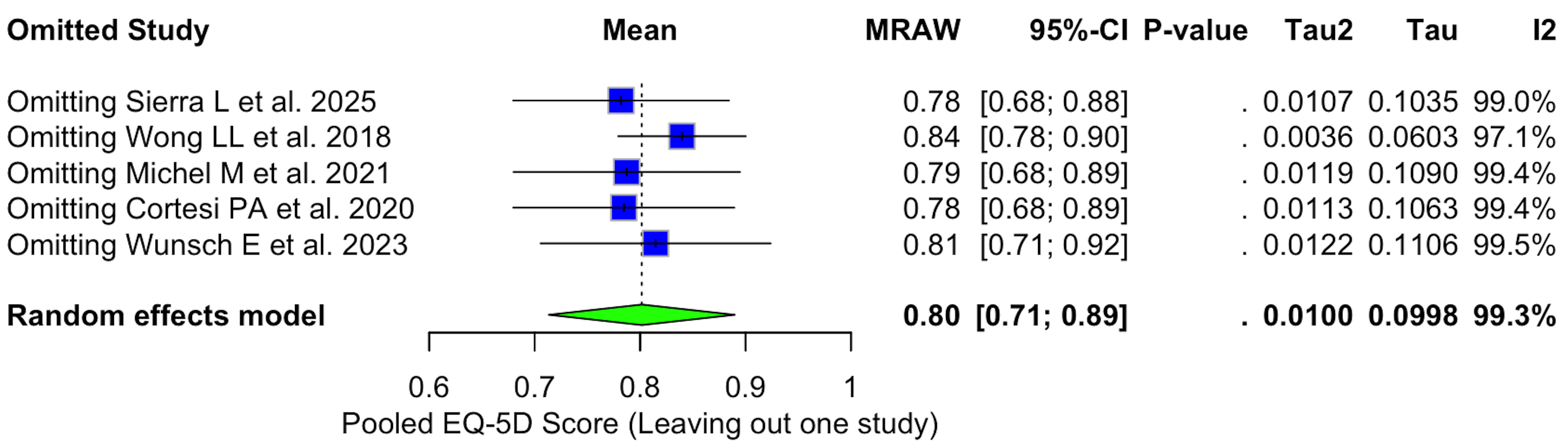 |
| --- |
| **Supplementary Figure 4. Leave-one-out sensitivity analysis of pooled EQ-5D utility scores in adults with autoimmune hepatitis.** |

| 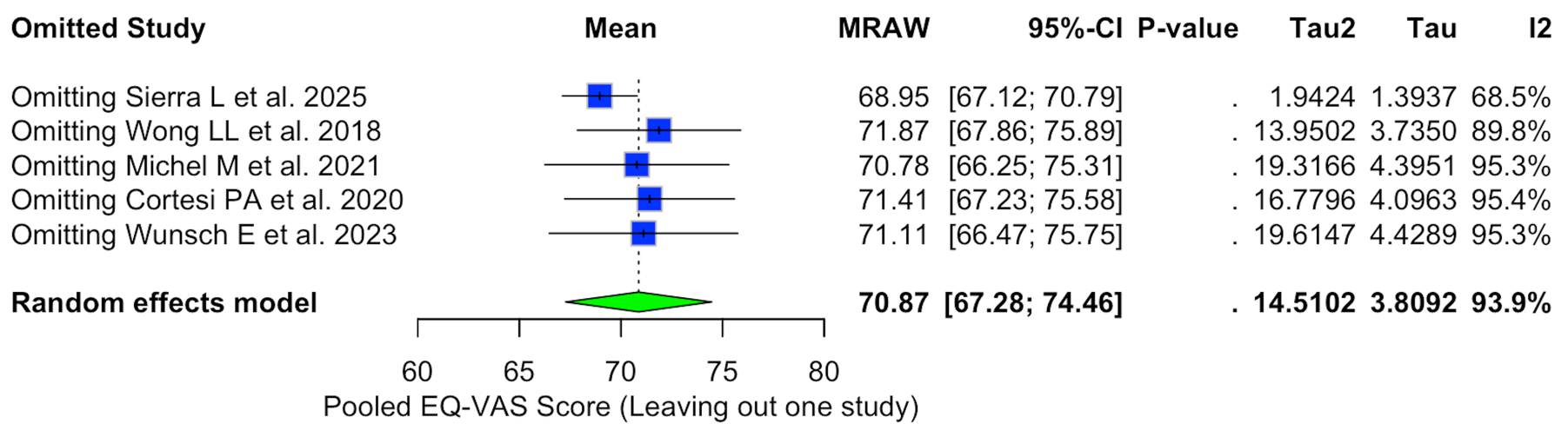 |
| --- |
| **Supplementary Figure 5. Leave-one-out sensitivity analysis of pooled EQ-VAS scores in adults with autoimmune hepatitis.** |

| **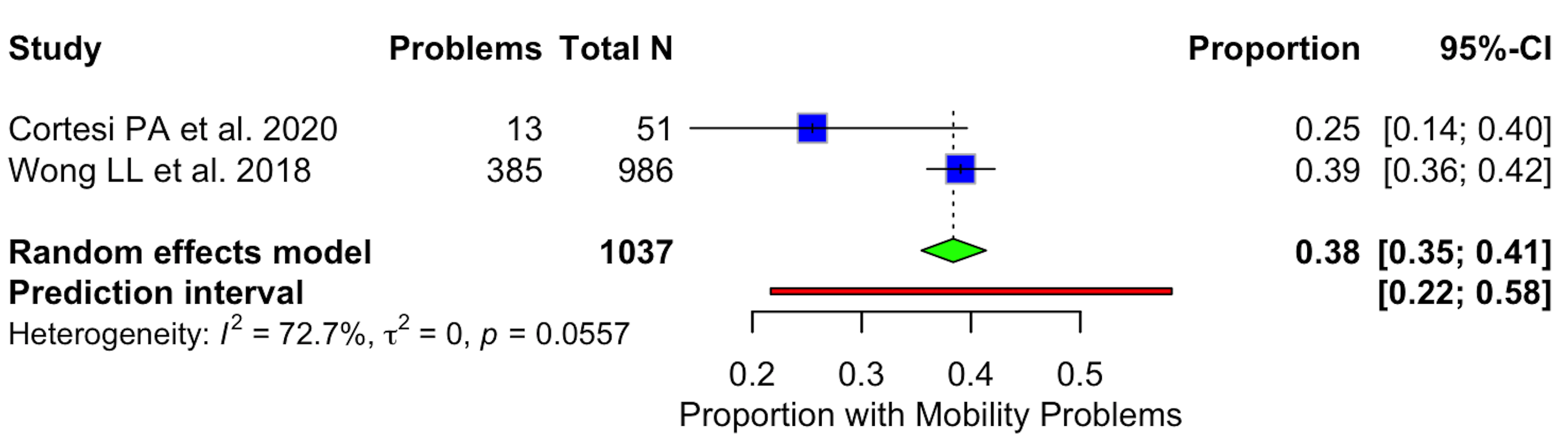** |
| --- |
| **Supplementary Figure 6. Forest plot of the pooled proportion of adults with autoimmune hepatitis reporting mobility problems based on the EQ-5D across included studies.** |

| **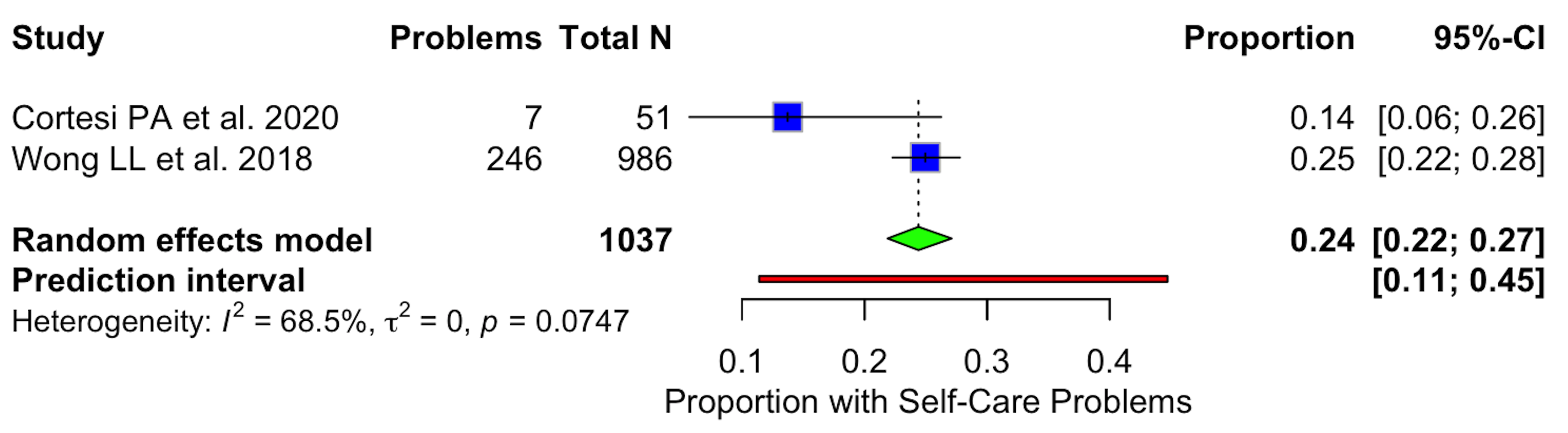** |
| --- |
| **Supplementary Figure 7. Forest plot of the pooled proportion of adults with autoimmune hepatitis reporting self-care problems based on the EQ-5D across included studies.** |

| **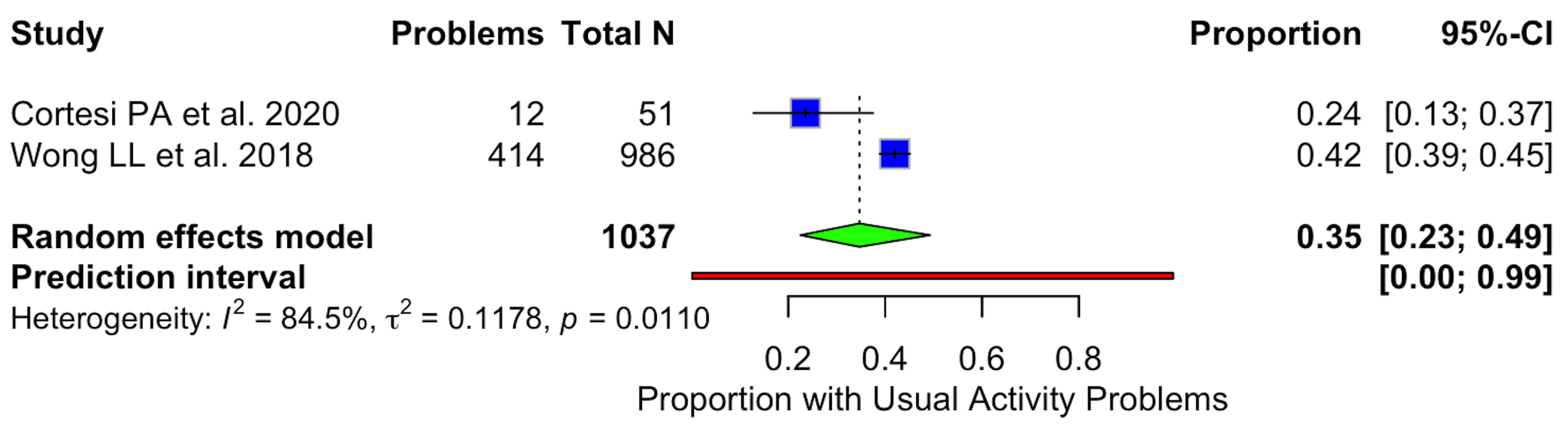** |
| --- |
| **Supplementary Figure 8. Forest plot of the pooled proportion of adults with autoimmune hepatitis reporting usual activities problems based on the EQ-5D across included studies.** |

| **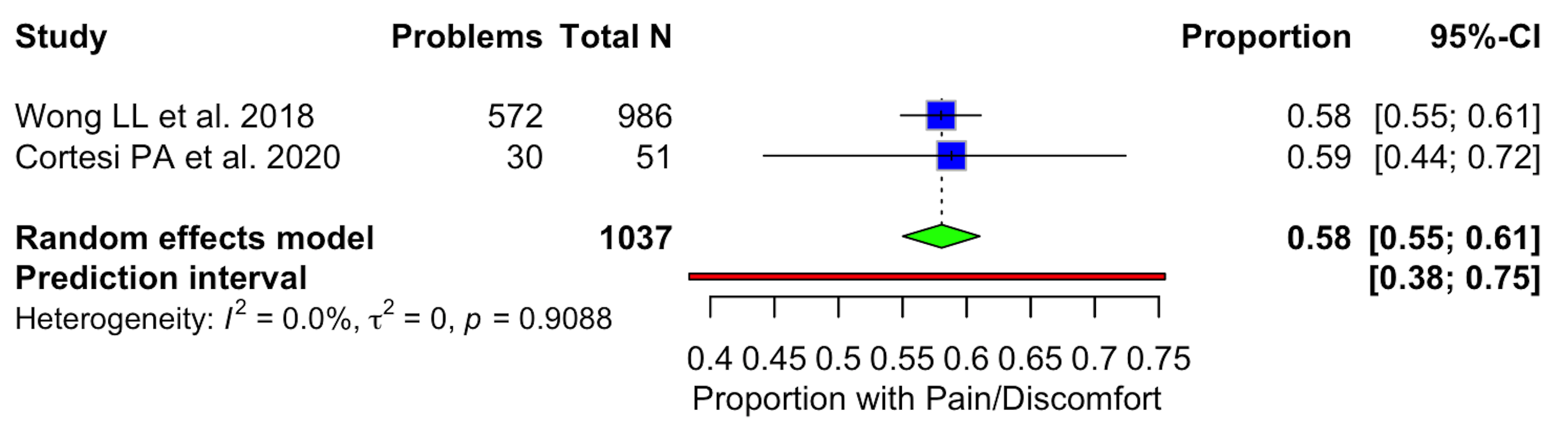** |
| --- |
| **Supplementary Figure 9. Forest plot of the pooled proportion of adults with autoimmune hepatitis reporting pain/discomfort problems based on the EQ-5D across included studies.** |

| **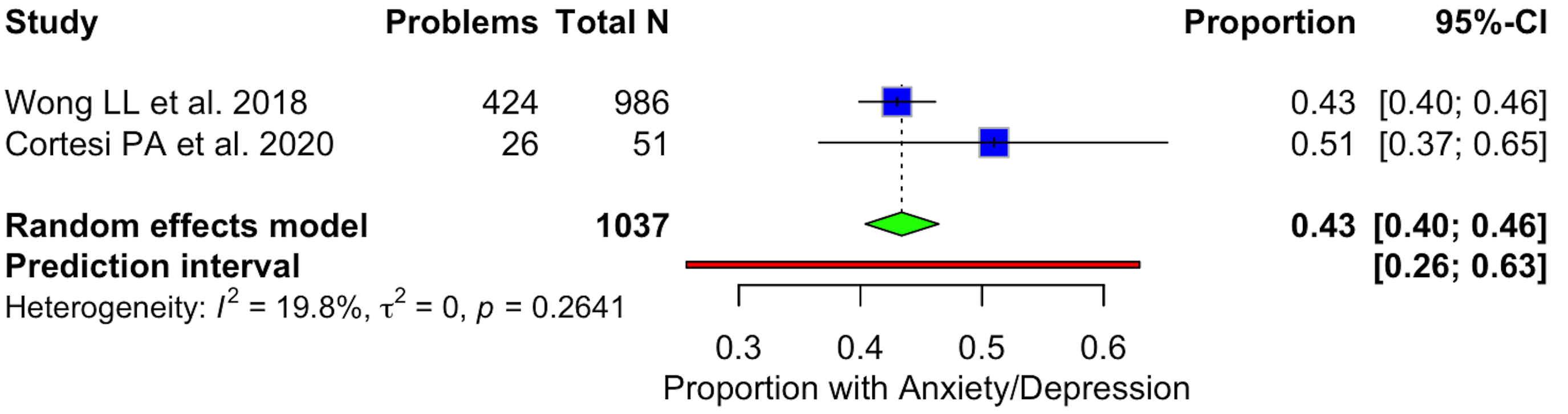** |
| --- |
| **Supplementary Figure 10. Forest plot of the pooled proportion of adults with autoimmune hepatitis reporting anxiety/depression problems based on the EQ-5D across included studies.** |

| **Outcome** | **Studies / participants** | **Pooled estimate** | **Risk of bias** | **Inconsistency** | **Indirectness** | **Imprecision** | **Publication bias** | **Overall certainty** |
| --- | --- | --- | --- | --- | --- | --- | --- | --- |
| **Overall EQ-5D utility** | 5 observational studies, 1,853 participants | 0.80 (95% CI; 0.71 to 0.89), I² = 99.3%;  PI 0.50 to 1.11 | Serious | Very serious | Not serious | Not serious | Not assessable (no downgrade) | **Very low** |
| **Overall EQ-VAS** | 5 observational studies, 1,852 participants | 70.87 (95% CI; 67.28 to 74.46), I² = 93.9%;  PI 59.14 to 82.61 | Serious | Very serious | Not serious | Not serious | Not assessable (no downgrade) | **Very low** |
| **EQ-5D domain proportions** | 2 observational studies, 1,037 participants | **Mobility** - 0.38 (0.35 to 0.41), I² = 72.7% **Self-care** - 0.24 (0.22 to 0.27), I² = 68.5%  **Usual activities** - 0.35 (0.23 to 0.49); I² = 84.5%, **Pain/discomfort** - 0.58 (0.55 to 0.61); I² = 0.0% **Anxiety/depression** - 0.43 (0.40 to 0.46), I² = 19.8% | Serious | Serious | Not serious | Serious | Not assessable (no downgrade) | **Very low** |

**Supplementary Figure 11. GRADE evidence profile for the meta-analyzed outcomes of EQ-5D in adults with autoimmune hepatitis, including overall EQ-5D utility, overall EQ-VAS, and domain-level pooled proportions.**

| **Section and Topic** | **Item #** | **Checklist item** | **Location where item is reported** |
| --- | --- | --- | --- |
| **TITLE** | | |  |
| Title | 1 | Identify the report as a systematic review. | 1 |
| **ABSTRACT** | | |  |
| Abstract | 2 | See the PRISMA 2020 for Abstracts checklist. | 2 |
| **INTRODUCTION** | | |  |
| Rationale | 3 | Describe the rationale for the review in the context of existing knowledge. | 3,4 |
| Objectives | 4 | Provide an explicit statement of the objective(s) or question(s) the review addresses. | 3,4 |
| **METHODS** | | |  |
| Eligibility criteria | 5 | Specify the inclusion and exclusion criteria for the review and how studies were grouped for the syntheses. | 4 |
| Information sources | 6 | Specify all databases, registers, websites, organisations, reference lists and other sources searched or consulted to identify studies. Specify the date when each source was last searched or consulted. | 4 |
| Search strategy | 7 | Present the full search strategies for all databases, registers and websites, including any filters and limits used. | 4, Supp Table 1-4 |
| Selection process | 8 | Specify the methods used to decide whether a study met the inclusion criteria of the review, including how many reviewers screened each record and each report retrieved, whether they worked independently, and if applicable, details of automation tools used in the process. | 4,5 |
| Data collection process | 9 | Specify the methods used to collect data from reports, including how many reviewers collected data from each report, whether they worked independently, any processes for obtaining or confirming data from study investigators, and if applicable, details of automation tools used in the process. | 5 |
| Data items | 10a | List and define all outcomes for which data were sought. Specify whether all results that were compatible with each outcome domain in each study were sought (e.g. for all measures, time points, analyses), and if not, the methods used to decide which results to collect. | 6 |
|  | 10b | List and define all other variables for which data were sought (e.g. participant and intervention characteristics, funding sources). Describe any assumptions made about any missing or unclear information. | 5 |
| Study risk of bias assessment | 11 | Specify the methods used to assess risk of bias in the included studies, including details of the tool(s) used, how many reviewers assessed each study and whether they worked independently, and if applicable, details of automation tools used in the process. | 5,6 |
| Effect measures | 12 | Specify for each outcome the effect measure(s) (e.g. risk ratio, mean difference) used in the synthesis or presentation of results. | 6,7 |
| Synthesis methods | 13a | Describe the processes used to decide which studies were eligible for each synthesis (e.g. tabulating the study intervention characteristics and comparing against the planned groups for each synthesis (item #5)). | 5,6 |
|  | 13b | Describe any methods required to prepare the data for presentation or synthesis, such as handling of missing summary statistics, or data conversions. | 6 |
|  | 13c | Describe any methods used to tabulate or visually display results of individual studies and syntheses. | 10 |
|  | 13d | Describe any methods used to synthesize results and provide a rationale for the choice(s). If meta-analysis was performed, describe the model(s), method(s) to identify the presence and extent of statistical heterogeneity, and software package(s) used. | 7 |
|  | 13e | Describe any methods used to explore possible causes of heterogeneity among study results (e.g. subgroup analysis, meta-regression). | 7 |
|  | 13f | Describe any sensitivity analyses conducted to assess robustness of the synthesized results. | 6 |
| Reporting bias assessment | 14 | Describe any methods used to assess risk of bias due to missing results in a synthesis (arising from reporting biases). | 6 |
| Certainty assessment | 15 | Describe any methods used to assess certainty (or confidence) in the body of evidence for an outcome. | 6 |
| **RESULTS** | | |  |
| Study selection | 16a | Describe the results of the search and selection process, from the number of records identified in the search to the number of studies included in the review, ideally using a flow diagram. | 7,8, Figure 1 |
|  | 16b | Cite studies that might appear to meet the inclusion criteria, but which were excluded, and explain why they were excluded. | 7, Supp Table 5 |
| Study characteristics | 17 | Cite each included study and present its characteristics. | 10, Table 1 |
| Risk of bias in studies | 18 | Present assessments of risk of bias for each included study. | 10, Supp Figure 1, 2, 3, 4 |
| Results of individual studies | 19 | For all outcomes, present, for each study: (a) summary statistics for each group (where appropriate) and (b) an effect estimate and its precision (e.g. confidence/credible interval), ideally using structured tables or plots. | 10,11 |
| Results of syntheses | 20a | For each synthesis, briefly summarise the characteristics and risk of bias among contributing studies. | 10 |
|  | 20b | Present results of all statistical syntheses conducted. If meta-analysis was done, present for each the summary estimate and its precision (e.g. confidence/credible interval) and measures of statistical heterogeneity. If comparing groups, describe the direction of the effect. | 10-14, Figure 2 & 3, Supp Figure 5 - 12 |
|  | 20c | Present results of all investigations of possible causes of heterogeneity among study results. | 12, 13 |
|  | 20d | Present results of all sensitivity analyses conducted to assess the robustness of the synthesized results. | 11,13, Supp. Fig 5,9 |
| Reporting biases | 21 | Present assessments of risk of bias due to missing results (arising from reporting biases) for each synthesis assessed. | 12, Supp. Fig 1,2 |
| Certainty of evidence | 22 | Present assessments of certainty (or confidence) in the body of evidence for each outcome assessed. | Supp. Fig 11 |
| **DISCUSSION** | | |  |
| Discussion | 23a | Provide a general interpretation of the results in the context of other evidence. | 13 |
|  | 23b | Discuss any limitations of the evidence included in the review. | 15 |
|  | 23c | Discuss any limitations of the review processes used. | 15 |
|  | 23d | Discuss implications of the results for practice, policy, and future research. | 15,16 |
| **OTHER INFORMATION** | | |  |
| Registration and protocol | 24a | Provide registration information for the review, including register name and registration number, or state that the review was not registered. | 4 |
|  | 24b | Indicate where the review protocol can be accessed, or state that a protocol was not prepared. | 4 |
|  | 24c | Describe and explain any amendments to information provided at registration or in the protocol. | 4 |
| Support | 25 | Describe sources of financial or non-financial support for the review, and the role of the funders or sponsors in the review. | 16 |
| Competing interests | 26 | Declare any competing interests of review authors. | 16 |
| Availability of data, code and other materials | 27 | Report which of the following are publicly available and where they can be found: template data collection forms; data extracted from included studies; data used for all analyses; analytic code; any other materials used in the review. | 16 |

**Supplementary Figure 12: PRISMA Checklist**
